# The History and Current Status of Platelet-Rich Plasma Therapy in Dermatology

**DOI:** 10.7759/cureus.68306

**Published:** 2024-08-31

**Authors:** Melanie Rodriguez, Harvey N Mayrovitz

**Affiliations:** 1 Osteopathic Medicine, Dr. Kiran C. Patel College of Osteopathic Medicine, Nova Southeastern University, Davie, USA; 2 Medical Education, Dr. Kiran C. Patel College of Allopathic Medicine, Nova Southeastern University, Davie, USA

**Keywords:** scars, prp, wounds, hypopigmentation, hyperpigmentation, wrinkles, ulcers, melasma, platelet rich plasma, dermatology

## Abstract

Since the 1800s, platelet-rich plasma (PRP) has been used as a treatment for a wide range of medical conditions with a concomitant effect of tending to reduce the need for some invasive procedures. The aim of this narrative review was to concisely document the history and current usage of PRP specifically in the field of dermatology. Four databases (PubMed, Google Scholar, CINAHL, and Web of Science) were searched for primary articles written in English that evaluated human subjects and focused on PRP use in dermatology. Initial search terms included “platelet rich plasma,” “alopecia,” “androgenic alopecia,” “dermatology,” “PDGF,” “aging,” “skin rejuvenation,” “diabetic ulcers,” “venous leg ulcers,” “acne,” “acne scars,” “scars,” “hyperpigmentation,” “melasma,” “hypopigmentation,” “vitiligo,” and “PRP.” After review, articles were excluded if they were commentaries, editorials, animal studies, review articles, or were unrelated to dermatology. The bibliography of retrieved articles was also searched for relevant articles. The present review results describe the function of PRP from its first usage for thrombocytopenia to its usage for melasma. In this time frame, its use in dermatology has gone through many evolutions from using its healing factors for treating wounds to using it as the treatment for wrinkles, hair loss, scars, ulcers, and skin pigmentation disorders. Its anti-inflammatory and growth factors have been shown to initiate a healing cascade that promotes the growth and regeneration of tissues. It is hoped that this review will help educate patients and physicians about the efficacy of PRP therapy and thereby help avoid unnecessary invasive procedures for certain conditions.

## Introduction and background

Early platelet therapy

The discovery of platelets began with the research of Max Schultze in 1865, where he provided a description of the constituents of white blood cells [1]. In 1883, Giulio Bizzozero studied the function of platelets in blood, publishing an accurate explanation of the important role that platelets play in the coagulation cascade [2]. In spite of the fact that the first transfusion of human blood into a human was reported in 1818 by James Blundell, it was not until 1910 that W.W Duke showed that a transfusion of platelet-rich plasma (PRP) into a young man with a very low platelet count was an effective treatment for thrombocytopenia [3,4]. Although the transfusion was a success, it was not until 1969 that Scott Murphy and Frank Gardner described an adequate storage method for platelets without losing the platelet’s hemostatic function [5]. Coagulation studies have shown a direct relationship between platelet transfusions and the improvement of thrombocytopenia and hemorrhagic episodes in patients with acute leukemia [6]. 

PRP is acquired from the patient’s own blood sample and then centrifuged to separate the red blood cells from the platelets, thereby concentrating the platelets in the plasma. In the 1970s, platelet-derived growth factors (PDGF) were shown to have growth-promoting activity in human glial cells and to accelerate the wound-healing cascade by causing a release of bioactive substances that impact tissue repair and cell proliferation [7,8]. In 1975, Oon and Hobbs described the clinical applications of PRP and later in 1987, PRP was used in heart surgery to avoid excessive blood transfusions [9,10]. Oon and Hobbs described how PRP was used as an adjunctive therapy in hypogammaglobulinemia, an immune-deficient disease, which reduced the risk of opportunistic infections from invading the patients [9]. In 1999, PRP began to be used for bone regeneration in dental usage [11]. In that study, PRP was used after dental implant surgery and showed a reduction in bone regeneration and soft tissue healing time. 

Initiation into dermatology

The use of PRP in the field of dermatology was initiated in 1986 to stimulate wound healing in chronic wounds [12]. That study showed that 48% of the patients treated with PRP had complete wound healing, compared to the placebo group in which only 14% of the patients had complete wound healing. PRP use has expanded into the field of dermatology wherein it is now being used for alopecia, ulcers, skin rejuvenation, acne, hypertrophic scars, hyperpigmentation, and hypopigmentation. The goal of this narrative review is to further document, characterize, and consolidate the history, current usage, contraindications, and implications of PRP in these dermatological treatments. 

## Review

Methods

The information presented in the following was obtained by the usage of four databases: PubMed, Google Scholar, CINAHL, and Web of Science. Searches were initiated using the keywords such as “platelet rich plasma,” “alopecia,” “androgenic alopecia,” “dermatology,” “PDGF”, “aging,” “skin rejuvenation,” “diabetic ulcers,” “venous leg ulcers,” “acne,” “acne scars,” “scars,” “hyperpigmentation,” “melasma,” “hypopigmentation,” “vitiligo,” and “PRP” within the text. After evaluating the articles from the four databases, the duplicate articles were excluded, and an inclusion criterion was used. Research studies were excluded if they were commentaries, editorials, animal studies, review articles, and were unrelated to the field of dermatology. The bibliography of the retrieved articles was also searched and assessed to check for eligibility based on the inclusion criteria. 

Venous ulcers

Venous ulcers are often the result of lower extremity venous valve dysfunction [13,14]. A failure of these valves to function properly initiates an event cascade that may result in skin ulceration with changes in blood flow and skin ulcer development, often on the lower leg [15,16]. The valve failure results in an elevated pressure in the veins ultimately causing the damage leading to the observed swelling and superficial erythematous ulcers [17]. The usage of PRP for the treatment of these chronic cutaneous ulcers was first researched in 1986 in an uncontrolled study [12]. The study showed that 49 patients with cutaneous ulcers, from different etiologies, were previously treated with conventional wound care for about 198 weeks without complete healing. After these same patients received platelet-derived wound healing factors, a combination of PDGF, platelet-derived angiogenesis factor, TGF beta, and platelet factor IV, there was 100% healing in 10.6 weeks [18]. In 2014, an uncontrolled study researched the usage of PRP in chronic nonhealing ulcers of various etiologies [19]. The study consisted of 24 patients who had 33 nonhealing ulcers. The results showed 91.7% and 95% reductions in the area and volume of the ulcers, respectively [19]. Out of the 33 ulcers treated, about 100% resolution was seen in 25 of the ulcers. 

Diabetic ulcers 

Diabetic ulcers are a complication that results from uncontrolled blood sugar levels and associated skin water and blood flow changes leading mostly to wounds of various sizes on the foot [20-23]. The efficacy of treating diabetic ulcers with PRP was first presented in 1995 [24]. In that study, PRP was compared to placebo as a treatment for chronic diabetic ulcers. The inclusion criteria for the study were patients with chronic, thickened, lower extremity diabetic ulcers which had been present for at least eight weeks. The ulcers treated were measured to be in the range of one to 100 cm^2^, with only one ulcer measuring 111 cm^2^ and 13 smaller than 1 cm^2^. The study consisted of a total of 118 patients, 61 patients were treated with PRP, and 57 were placed in the placebo group. The group that used the PRP showed that 48% of the patients achieved wound healing versus 25% of the placebo group. In 1998, Wieman and his coworkers studied the usage of a gel from recombinant human PDGF in the treatment of diabetic ulcers [25]. The study showed that compared to the placebo group (N=127), the usage of topical PDGF increased the rate of complete wound closure by decreasing the healing time from 127 days to 86 days, a 32% reduction. Out of the 123 patients treated with PDGF, 61 patients reached complete wound healing while only 44 patients from the placebo group reached complete wound healing; 50% and 35% complete wound healing were reached, respectively. In 2022, an observational study showed higher rates of wound reduction in diabetic foot ulcers for those treated with PRP than those treated with conventional dressings, the control group [26]. The results showed that PRP was efficient in healing the wound in 80% of the patients, 64 out of 80 patients, compared to the control group (N=80), which only showed improvement in 46.25% of the patients. 

Alopecia

Androgenic alopecia (AGA) is a genetically predetermined condition that causes an excessive production of dihydrotestosterone (DHT) [27]. DHT is an androgen that when produced in excess causes a reduction in the diameter, length, and density of the hair [27]. The usage of PRP in alopecia was first studied in 2006 by Uebel. In that study, he researched the effect of storing follicular units in PRP before hair graft surgery [28]. In this study, the hair grafts that were stored in PRP had a 15.1% increase in follicular density compared to the control group. In 2012, the effects of PRP were studied at a microscopic level. This study showed that PRP increased the number of dermal papilla cells, extracellular regulated kinase, Akt signaling, fibroblast growth factor 7, and beta-catenin, all of which stimulate hair growth [29]. In 2018, the usage of PRP for the treatment of AGA in Asian males also showed improvement in hair growth [30]. The study indicated that 96% of the patients treated with PRP reported satisfaction after six months and 80% of the patients reported satisfaction after one year post-treatment. 

PRP has also been shown to treat alopecia areata. Alopecia areata is an autoimmune disease that attacks the hair follicles causing non-scarring alopecia through its inflammatory process [31]. Several of these studies did not use a control group and are referred to as uncontrolled. An uncontrolled study performed in 2015 showed that the usage of PRP in patients with alopecia areata showed improvement in hair growth at the end of the six-month treatment [31]. Out of the 20 patients who were treated in that study, only one patient had a hair loss relapse with minimal hair growth [31]. In 2020, an uncontrolled study showed the usage of PRP to treat alopecia areata barbae, a type of alopecia areata that only affects the beard area [32]. The patient was given three PRP injections within a six-week period. The results showed a significant increase in hair regrowth after the one-year follow-up appointment. Recently, in 2023 a case report was published about a 46-year-old patient with severe alopecia areata [33]. These uncontrolled studies indicate that treatments with PRP stimulated hair regrowth and improved the patient’s standard of living. 

Skin rejuvenation 

Aging is an inevitable process that causes changes in the structures of the layers of the skin [34]. As time progresses, the epidermis and the dermal-epidermal junction become thinner and flatter, increasing skin fragility and resulting in changes in skin firmness and hydration in both sexes [34-37]. The first documented usage of PRP in face and neck revitalization was in an uncontrolled clinical series in 2010 [38]. The study encompassed 23 patients treated with PRP monthly for three months. The results were measured by imaging and patient satisfaction score, which showed improvement and high patient satisfaction. In 2011, Kim and coworkers studied the effects of aPRP and platelet-poor plasma (aPPP) and the expression of collagen and cell proliferation [39]. PRP and aPRP are the same compounds with the only difference being that aPRP is activated before, which leads to the immediate release of growth factors [39]. The results showed that both aPRP and aPPP stimulated cell proliferation; however, the group treated with aPRP had the highest cell proliferation. The protein expression of type I collagen was significantly higher in the aPRP group. The authors concluded that aPRP was an effective treatment for skin rejuvenation due to its stimulating factors. 

In 2012, Scarano and co-investigators studied the efficacy of treating rhytids (wrinkles) with PDGF to evaluate if the treatment achieved full face skin rejuvenation [40]. This uncontrolled trial showed that all 16 patients showed improvement and most of the patients (92.88%) who were treated with PRP were very satisfied with their results. Subsequently, in 2017, an uncontrolled study was conducted to test the efficacy of PRP as a treatment for facial wrinkles [41]. To evaluate the results, the authors used a rating scale known as the Wrinkle Severity Rating Scale (WSRS) [41]. The WSRS is a valid instrument that uses a five-grade scale to test for facial skin folds [42]. They reported an average scale score reduction from 2.90 to 2.10 after 20 patients were treated with eight weeks of PRP intradermal injections. The patients that showed the best improvement were the younger patients whose initial wrinkles were mild to moderate [41].

Acne and hypertrophic scars 

Acne scars are post-inflammatory lesions associated with acne vulgaris that have an increased risk of occurring attributable to touching and picking of acne [43]. PRP was first used for acne scars in 2010 by Fabbrocini and co-researchers [44]. In this study, 12 patients with acne scars had one-half of their face treated with microneedling and PRP while the other side was just treated with microneedling. The results were monitored with photographs and the signs test, which showed if there was a statistically significant difference between the two groups [44]. The photographs showed improvement in the entire face, but the severity of the scars significantly improved the most on the side of the face which was treated with PRP. In 2011, a split-face trial was conducted that studied the effect of PRP on acne scars [45]. In this study, the entire face was treated with ablative carbon dioxide fractional but only half the face was treated with PRP with the other half receiving normal saline. The results were based on the residual erythema and edema, which were measured objectively and with a chromometer. Both the edema and erythema of the face resolved faster in the group treated with PRP. Four months after the treatment there was also more clinical improvement in the acne scars treated with PRP. 

In 2019, Deshmukh and co-workers conducted a study that tested the usage of PRP with subcision versus subcision alone for acne scar treatment [46]. Subcision is a subcutaneous incisionless type of surgery [47]. The results of this study showed that the area treated with PRP had a greater improvement compared to the area treated with subcision alone [46]. Recently, in 2021, Nandini conducted a study that retested the usefulness of combining PRP with microneedling versus microneedling alone in 30 patients for the treatment of post-acne scars [48]. In the study, both groups showed improvement in acne scarring; however, 93% (28 patients) in the group treated with PRP and microneedling showed improvement while 73% (22 patients) in the group using microneedling alone improved; however, the differences in outcomes were not statistically significant. In a more recent study, Yadav and colleagues evaluated the use of PRP with either subcision or microneedling [49]. The study consisted of 50 patients with post-acne facial scars. In this spilt face study, the left side of the face of the patients was treated with microneedling and PRP while the right side was treated with subcision and PRP. The results were measured using the Goodman and Baron qualitative and quantitative grading. The report showed statistically significant improvement in both modalities but no significant difference in outcomes between the two groups. Out of the 50 patients, 70% had two grades of improvements on the left side of the face while 50% of the patients had the same type of improvement on the right side. 

Acne scars are not the only type of scars that PRP has shown to improve. In 2012, a study was conducted to test the usage of PRP in striae distensae (stretch marks) [50]. The results of this uncontrolled study that evaluated 18 patients with stretch marks showed that the average width of the widest striae decreased from 0.75 mm to 0.27 mm. The data indicated that 72.2% of the patients were highly satisfied with their improvement. In 2017, an uncontrolled study evaluated the efficacy of using PRP as a treatment for keloids [51]. The study consisted of 50 ear keloids that were treated with a combination of excisional surgery, in-office superficial radiation therapy, and PRP. The results demonstrated a 94% nonrecurrence rate over a two-year period after the combined treatment. Even though the study shows a positive effect of the usage of PRP as a combined therapy for keloids, the study does not compare the combined therapy without the usage of PRP. Therefore, the study tells nothing about the effect of PRP on keloids. In 2022, Hewedy and colleagues studied the effectiveness of triamcinolone versus triamcinolone and PRP for the treatment of keloids [52]. The progress was quantified using the Vancouver scar scale (VSS) and verbal rating scale (VRS). Both groups showed improvement, but the group treated with PRP had greater improvement in the height, pigmentation, and consistency of the keloids. It was also noted that the addition of PRP to the treatment regimen decreased the incidence of skin atrophy and hypopigmentation induced by triamcinolone. 

Hypertrophic scars have also been treated with PRP. These scars are associated with excessive production of fibroblasts, which is driven by high levels of TGF‐β1 [53]. In 2018, a study was conducted that evaluated the effect of PRP on hypertrophic scars. The study showed that using PRP as therapy for hypertrophic scars seems to cause a negative feedback loop, increasing TGF‐β1 and decreasing CTGF levels leading to diminishing scar formation [53].

Hyperpigmentation and hypopigmentation

Melasma is a hyperpigmentation skin condition that generally affects sun-exposed skin often the face of women who have Fitzpatrick skin phototype 3-6 [54]. There are multiple factors, apart from sun exposure, that could trigger melasma on the skin including pregnancy, oral contraceptives, genetics, cosmetics, and race [55]. The usage of PRP in hyperpigmentation was first studied in 2014 for the treatment of infraorbital dark circles and crow’s feet wrinkles [56]. The study demonstrated a significant improvement in infraorbital color homogeneity after using PRP as a treatment. In 2019, Hofny and co-workers studied the expression levels of TGF-β protein in melasma patches after treating the area with PRP [57]. TGF-β is a protein that induces the healing of hyperpigmentation by reducing melanin synthesis. The research consisted of a group with healthy skin (N=9) and a group that suffered from melasma (N=11). Initially, before any treatment was given, the patients with melasma had significantly lower levels of TGF-β compared to the healthy group. After the treatment of PRP, the TGF-β levels increased from 1.26 to 2.15 in the lesional skin of melasma, with the healthy group having a TGF-β expression of 2.26.

In 2021, Mumtaz and colleagues compared the efficacy of PRP versus tranexamic acid as a 24-week treatment of 64 patients with melasma [58]. The treatment outcome was assessed using the melasma severity index score (MASI). This index was developed to quantify the severity of melasma [59]. The score is calculated by a subjective assessment. The area evaluated is given a value from zero to six depending on the percentage of the area involved, with zero being the absence of melasma to six being the maximum [59]. Darkness and homogeneity are also calculated using a similar scale; the scores are then put into a formula to calculate the MASI total score [59]. The average starting MASI score for the group treated with PRP and the group treated with tranexamic acid was 29.84 and 29.56, respectively. After 24 weeks of treatment, the average MASI was 8.72 for those treated with PRP and 14.97 for those treated with tranexamic acid [58]. The comparison between PRP and tranexamic acid was subsequently evaluated by using a split face design in 2023 by Elraouf and colleagues [60]. The mean starting MASI score was 4.72 and 4.59, respectively. After three months of treatment, the MASI score reduced by an average of 53.66% for the group treated with PRP versus 45.67% for the group treated with tranexamic acid. 

Vitiligo is a skin disorder that presents as hypopigmented patches throughout the body’s skin [61]. The exact etiology of vitiligo is unknown but is thought to be an autoimmune disease that attacks the melanocytes [62]. PRP was first studied to be used as a treatment for vitiligo in 2016 by Ibrahim and coworkers [63]. The study was set to treat patients with symmetrical vitiligo lesions with narrowband ultraviolet B (NB-UVB) and PRP. For each patient, one side of the body was treated just with NB-UVB and the other side was treated both with NB-UVB and PRP. Both treatments showed to have an improvement in the treatment of vitiligo. The results, however, showed a greater improvement in repigmentation in the areas treated with the combination of PRP and NB-UVB compared to the group just treated with NB-UVB. In 2019, Parambath et al. studied the usage of PRP together with the transplantation of epidermal cells for patients with vitiligo [64]. The transplantation procedure had been shown to be effective in treating vitiligo, but the addition of PRP improved the rate of healing and repigmentation in vitiligo patches. In this study, the patients were re-evaluated six months after the procedure and the average repigmentation in the areas treated with PRP was 75.6 cm^2^ compared to 65 cm^2^, which was the value for the areas not treated with PRP. 

## Conclusions

The present review results describe the function of PRP from its first usage for thrombocytopenia to its usage for melasma. In this time frame, its use in dermatology has gone through many evolutions from using its healing factors for treating wounds to using it as a treatment for wrinkles, hair loss, scars, ulcers, and skin pigmentation disorders. Its anti-inflammatory and growth factors have been shown to initiate a healing cascade, which promotes growth and regeneration of tissues. It is hoped that this review helps educate patients and physicians about the efficacy of PRP therapy to avoid unnecessary invasive procedures for the treatment of certain conditions.
